# Reactive oxygen species at the crossroads of inflammasome and inflammation

**DOI:** 10.3389/fphys.2014.00352

**Published:** 2014-09-29

**Authors:** Anantha Harijith, David L. Ebenezer, Viswanathan Natarajan

**Affiliations:** ^1^Department of Pediatrics, University of IllinoisChicago, IL, USA; ^2^Department of Biochemistry, University of IllinoisChicago, IL, USA; ^3^Department of Pharmacology, University of IllinoisChicago, IL, USA; ^4^Department of Medicine, University of IllinoisChicago, IL, USA

**Keywords:** inflammasome, inflammation, reactive oxygen species

## Abstract

Inflammasomes form a crucial part of the innate immune system. These are multi-protein oligomer platforms that are composed of intracellular sensors which are coupled with caspase and interleukin activating systems. Nod-like receptor protein (NLRP) 3, and 6 and NLRC4 and AIM2 are the prominent members of the inflammasome family. Inflammasome activation leads to pyroptosis, a process of programmed cell death distinct from apoptosis through activation of Caspase and further downstream targets such as IL-1β and IL-18 leading to activation of inflammatory cascade. Reactive oxygen species (ROS) serves as important inflammasome activating signals. ROS activates inflammasome through mitogen-activated protein kinases (MAPK) and extracellular signal-regulated protein kinases 1 and 2 (ERK1/2). Dysregulation of inflammasome plays a significant role in various pathological processes. Viral infections such as Dengue and Respiratory syncytial virus activate inflammasomes. Crystal compounds in silicosis and gout also activate ROS. In diabetes, inhibition of autophagy with resultant accumulation of dysfunctional mitochondria leads to enhanced ROS production activating inflammasomes. Activation of inflammasomes can be dampened by antioxidants such as SIRT-1. Inflammasome and related cascade could serve as future therapeutic targets for various pathological conditions.

## Introduction

Inflammation is a response of the body to tissue injury, and plays an essential role in tissue repair when regulated. Chronic inflammation is always pathological and a dysregulated inflammation results in significant tissue damage. Inflammation is induced by diverse stimuli, and is the outcome of a shift in balance between various pro and anti- inflammatory cytokines. Inflammasomes are newly discovered multi-protein oligomer platforms that include Nod-like receptor protein (NLRP) 3 and 6, NLR family CARD domain-containing protein (NLRC) 4 and Absent in Melanoma-2 (AIM2) protein. These play an important role in initiating and sustaining inflammation (Wu et al., 2010). The inflammasome is a component of the innate immune system, and comprises an intracellular sensor such as NLRPs which is coupled with procaspase-1 and the adaptor Apoptosis-associated speck-like protein containing a carboxy-terminal CARD, i.e., ASC. Activation of inflammasome complex triggers the maturation of caspase-1, resulting in the processing of interleukin 1β (IL-1β) and IL-18. IL-1β is the most active cytokine in acute lung injury patients (Ganter et al., 2008). The inflammasomes activate inflammation to induce cell pyroptosis, a process of programmed cell death different from apoptosis (Fink and Cookson, 2005), and the exact nature of inflammasome depends on the activating agent. Pathogen-Associated Molecular Patterns (PAMPs) that are predominantly of microbial origin and Damage-Associated Molecular Patterns (DAMPs) that are released following microbial or non-microbial tissue damage are two frequent stimulants of inflammasome that induce maturation of pro-inflammatory cytokines (Savage et al., 2012). NLRP3 inflammasome is probably the most widely studied and is activated by host-derived molecules such as excess ATP, glucose, reactive oxygen species (ROS), sphingosine, ceramides, oxidized LDL, uric acid, and crystals of cholesterol (Duewell et al., 2010; Jiang et al., 2012; Luheshi et al., 2012; Bandyopadhyay et al., 2013; Fukumoto et al., 2013).

Improper regulation of inflammasome production could adversely affect the balance between pro- and anti-inflammatory cytokines, leading to inflammation and pyroptosis. Though NLRP3 inflammasome is widely studied, little is known about the underlying molecular mechanisms that regulate its assembly and activation. Recent studies show that generated by NLRP3 activators, ROS acts as second messengers whose signaling drives inflammasome activation (Fukumoto et al., 2013; Heid et al., 2013). There have been several excellent review articles that have addressed inflammasomes, role of mitochondria in inflammasome activation, and inflammasomes in health and diseases. This review will specifically address the role ROS plays in inflammasome activation and its implication in the pathology of human diseases.

### NLR proteins and inflammasomes

Inflammasomes are classified into two families, viz., NLR and PYHIN (pyrin and hematopoietic interferon-inducible nuclear antigens domain-containing protein) (Fernandes-Alnemri et al., 2009; Broz and Monack, 2011). The NLR family has more than 20 members currently known in humans that include nucleotide-binding domain (NBD) and leucine-rich repeat (LRR) proteins such as NLRP1, NLRP2, NLRP3, NLRP6, NLRC4, and NLRP12 (Correa et al., 2012; Allen et al., 2013; Chen, 2014). The LRR domains of this family may be involved in auto-inhibition, recognition of PAMPs, and protein-protein interactions. The NBDs can bind ribonucleotides and possibly regulate homo- or hetero-oligomerization, required for inflammasome assembly. They also have a pyrin domain (PYD), a caspase activation and recruitment domain (CARD), or both. The PYHIN family members include AIM2 and Gamma-interferon-inducible protein (IFI16), and are characterized by a HIN200 ligand binding domain, in addition to the PYD that is involved in ligand binding.

### ROS in NLRP3 inflammasome activation

Recent studies suggest that ROS production is induced by many NLRP3 inflammasome stimulators, and elevated ROS is essential for inflammasome activation; however, the cellular source of ROS in mediating these responses remains unclear. ROS is primarily a byproduct of mitochondrial oxidative phosphorylation. During respiration, ~1–2% of molecular oxygen (O_2_) is partially reduced to superoxide and OH^·^, and the major sites of their production in the mitochondrial respiratory chain are at complex I and III. Complex I accepts electrons from NADH, which move down an electrochemical gradient through ubiquinone to complex III, then further down to cytochrome c and complex IV where O_2_ is reduced to water. In addition to the mitochondrial electron transport chain, ROS is also generated in mammalian cells by the activity of enzymes such as NADPH oxidases (NOXs), xanthine oxidase (XO), cyclooxygenase and lipoxygenases (Habu et al., 1990; Lacy et al., 1998; Andrew and Mayer, 1999; Paravicini and Touyz, 2008). There is compelling evidence that ROS production by NLRP3/NALP3 activators involves NOX family of transmembrane proteins. These proteins generate ROS by transporting electrons across biological membranes from a cytosolic donor (NADPH) to an electron acceptor, O_2_, in the extracellular or luminal space (Martinon, 2010; Nakahira et al., 2011). While ROS generation is essential for cell signaling and several important physiological responses, excessive accumulation of ROS can lead to cellular damage and death. To mitigate this oxidant stress, cells have several anti-oxidant defense mechanisms that include superoxide dismutase (SOD), glutathione peroxidase (GPX), thioredoxin, catalase (CAT) and peroxiredoxins (Rahman et al., 2006; Johnson et al., 2010). ROS production by polymorphonuclear leukocytes (PMN) and monocytes when exposed to priming by NLRP3 activators regulates the activation of redox-dependent transcriptional factors such as nuclear factor kappa-light-chain-enhancer of activated B cells (NF-kB) and activator protein 1 (AP-1) via Mitogen-activated protein kinases (MAP Kinases) and production of pro-inflammatory cytokines (Kabe et al., 2005; Bauernfeind et al., 2009).

**Figure 1:**
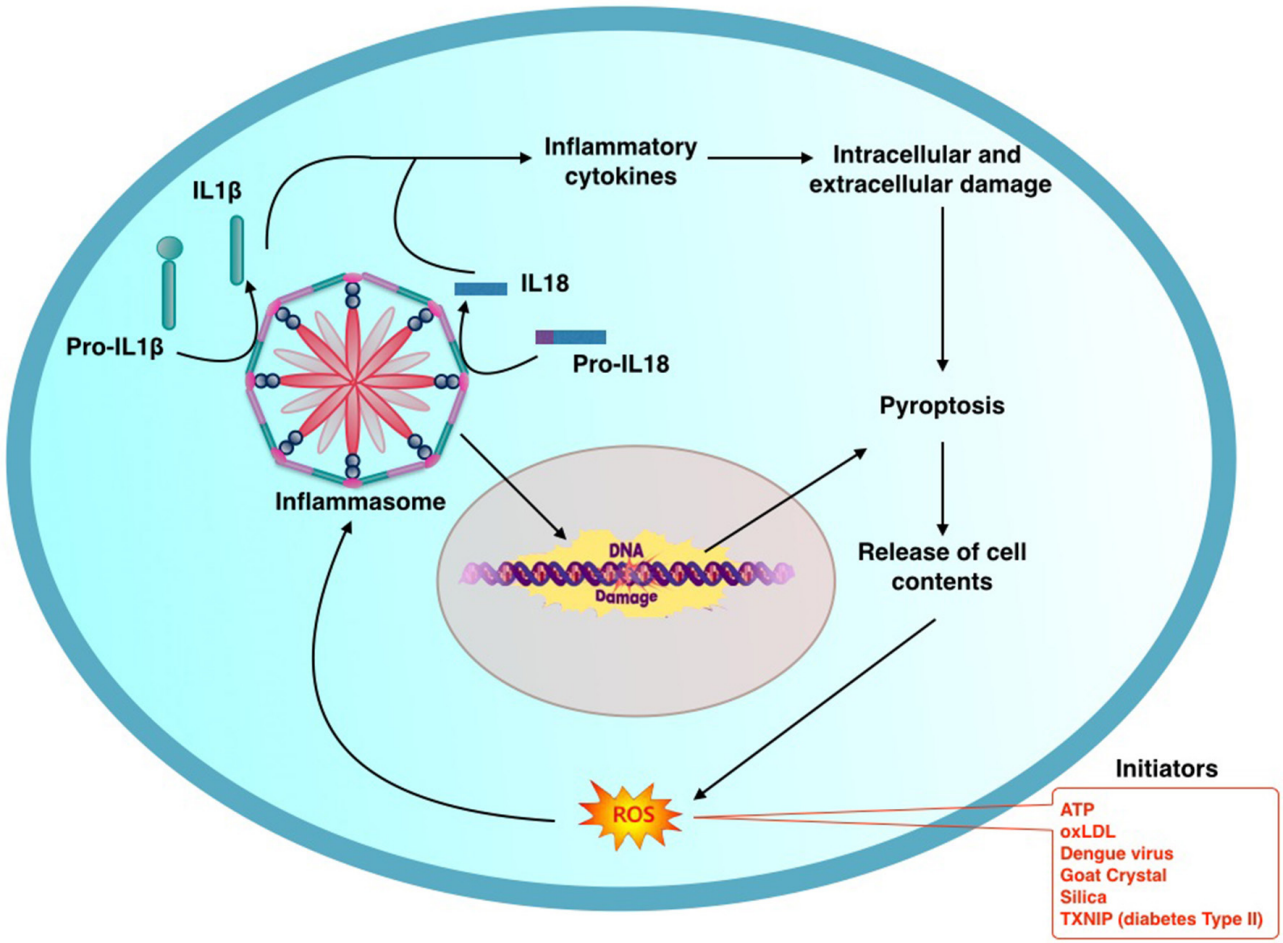
**General schema describing the process of activation of inflammasome: initiating factors activate production of reactive oxygen species (ROS) which in turntriggers the inflammasome mediated inflammatory cascade**. Oligomerization of components results in assembly of Inflammasome. This in turn activates Il-1β and Il-18 through caspase-1. NLRP3 Inflammasome promotes oxidative DNA damage. Inflammation and DNA damage culminates in pyroptosis releasing contents from the damaged cell. This in turn promotes a vicious cycle of further Inflammasome mediated pathogenic process.

Animal studies have shown that activation of IL-1β and caspase-1 is ROS-dependent (Dostert et al., 2008; Franchi et al., 2009). In a positive feedback loop, IL-1β promotes intracellular accumulation of ROS by uncoupling superoxide dismutase (SOD), glutathione peroxidase (GPX), and catalase (CAT). The NLRP3 inflammasome is known to play a critical role in caspase-1 activation and the proteolytic processing of pro-IL-1β (Figure 1). Activation of IL-1β and IL-18 inflammatory cytokines is independently regulated by NLRP3 inflammasome. This has been shown by using a “non-canonical” stimulus such as the secreted *Listeria monocytogenes* (Lm) p60 protein. Primed murine dendritic cells (DCs) respond to p60 stimulation by producing ROS and secreting IL-1β and IL-18 without any accompanying pyroptosis. Inhibitors of ROS production inhibit secretion of IL-1β, but not that of IL-18 (Schmidt and Lenz, 2012). Recent studies have demonstrated a role for CLOCK gene following activation of NALP3. A dose-dependent increase in CLOCK gene expression is noted with increased oxygen concentrations. A significant CLOCK gene down regulation occurs in *Nalp3^−/−^* mice compared to wild-type controls, suggesting its role downstream of NALP3 in mediating hyperoxia-induced lung inflammation (Lagishetty et al., 2014). Exact mechanism(s) that regulates NLRP3 inflammasome activation and its downstream activators resulting in pyroptosis still remains unclear.

NLRX1, a recently identified member of the NLR family, plays an active role in ROS production. In contrast to other NLR family members which are located in the cytosol, NLRX1 is specifically associated with the mitochondrial membrane and differs structurally from the other NLRs. NLRX1 shows structural homology with the NOD-subfamily. Inflammatory signals like TNF-α require NLRX1 for ROS production (Gavelli et al., 1990; Álvarez and Muñoz-Fernández, 2013). Importance of NLRX1 in caspase-1 activation and IL-1β signaling is yet to be characterized.

### Role of NOX proteins in NLRP3 inflammasome activation

Among a host of inflammation-inducing agents, a common convergence point that leads to activation of NLRP3 is yet to be identified. ROS could act as a common event upstream of the NLRP3 inflammasome machinery. NLRP3 is unique in that its activation is quite sensitive to ROS inhibition (Heid et al., 2013). NLRP3 inflammasome activation requires priming by pro-inflammatory signals whereas ROS inhibitors block the priming of NLRP3. Moreover, pre-treatment of cells with antioxidants inhibits NLRP3/NALP3 inflammasome activation, suggesting a potential role for oxidative stress/redox signaling in NLRP3/NALP3 activation (Bauernfeind et al., 2011; Hua et al., 2013). The role of ROS in inflammasome activation has become more evident as studies have shown that NLRP3-inflammasome activators like silica, asbestos and ATP require ROS production for their activity. In mammals, extracellular ATP binds to P2X7 receptors, induces rapid accumulation of ROS and activates the NLRP3/NALP3 inflammasome (Riteau et al., 2012). The source of ROS triggered by ATP seems to be NOX-derived, as the NOX inhibitor, diphenyleneiodonium (DPI) inhibits ROS-dependent ATP-mediated caspase-1 activation caused by ROS (Cruz et al., 2007). Exposure to silica is known to result in ROS generation in the bone marrow-derived macrophages from both wild type and *Nlrp3^−/−^* mice, suggesting that ROS generation is upstream of NLRP3 activation (Cassel et al., 2008). Similarly, uric acid crystals, alum, and particulate metals are known to activate NLRP3 via ROS production mediated by mitochondrial or NOX protein activation. However, other NLRP3/NALP3 activators such as nigericin (toxin) and UV light induce cellular redox imbalance that is necessary for inflammasome formation (Martinon, 2010). Interestingly, ROS has also been implicated in inflammasome activation by hemozoin (malaria pathogen crystal), the influenza virus and the yeast *Candida albicans* (Sakai et al., 1989; Shio et al., 2009; Ichinohe et al., 2010). High concentrations of calcium-binding Myeloid Related Proteins such as S100A8 and S100A9 promote phagocytosis and have been noted in inflammatory conditions. They promote NOX activation by interaction with p67phox and Rac-2 (Doussiere et al., 2002; Kerkhoff et al., 2005). S100A8 and S100A9-mediated activation of NF-κ B, the NLR family, pyrin domain-containing 3 (NLRP3) protein, and pro-IL-1β expression depend on the generation of ROS (Simard et al., 2013).

#### Role of mitochondrial ROS in NLRP3 inflammasome activation

While NOX proteins seem to be the main source of ROS in particle-induced phagocytosis and inflammasome activation, mouse cells deficient in NOX2 show diminished ROS production in response to various inflammasome activators, with a reduction in inflammasome activation (Abais et al., 2013). Additionally, neutrophils derived from patients with chronic granulomatous disease bearing mutation(s) in NOX2 display defective ROS production, but inflammasome activation is unaffected (Meissner et al., 2010) suggesting involvement of additional factors in regulating inflammasome activation. Recent evidence suggests a role for mitochondrial ROS in inflammasome activation independent of NOX proteins. Using techniques to modify mitochondrial function and uncouple the respiratory chain, Zhou et al., has demonstrated redistribution of both NLRP3 and its adaptor ASC to the perinuclear space where they co-localize with endoplasmic reticulum and mitochondria (Zhou et al., 2011). Further, dysregulation of mitochondrial activity by inhibition of the voltage-dependent anion channel suppresses ROS generation and inflammasome activation. Another salient feature of this study is a role for mitophagy in inflammasome activation. Inhibition of mitophagy/autophagy with 3-methyladenine results in accumulation of damaged mitochondria, increased mitochondrial ROS, and activation of inflammasome, suggesting that NLRP3 inflammasome may sense mitochondrial damage and dysfunction in inflammatory diseases (Zhou et al., 2011). IL-1β is recognized as a reliable marker for identifying ventilator-induced lung inflammation. Cyclic stretch is known to activate NLRP3 inflammasomes and mechanical ventilation activates the NLRP3 inflammasomes in mouse alveolar macrophages, increasing the production of IL-1β. IL-1β neutralization reduces mechanical ventilation-induced inflammatory lung injury. Activation of NLRP3 inflammasomes follows release of IL-1β in mouse alveolar macrophages via caspase-1 and TLR4-dependent mechanisms. It has been noted that mitochondrial generation of ROS is required for stretch-induced NLRP3 inflammasome activation and IL-1β release, whereas NOX2 is not essential (Kuipers et al., 2012; Wu et al., 2013a).

#### Modulation of NLRP3 inflammasome by tripartite-motif protein 30

Tripartite-motif family of proteins or TRIM proteins is involved in pathogen-recognition, and regulates transcriptional pathways in host defense. TRIM30 negatively regulates NLRP3 inflammasome activation. Knockdown of TRIM30 enhances caspase-1 activation and increases production of IL-1β in bone marrow-derived macrophages. TRIM30 knockdown increases ROS production, which enhances NLRP3 inflammasome activation. On the contrary, over-expression of TRIM30 attenuates ROS production, and suppresses NLRP3 inflammasome activation; however, the source of ROS has not been identified. *In vivo* experiments in a crystal-induced NLRP3 inflammasome-dependent peritonitis model using TRIM30 transgenic mice show significant reduction of monosodium urate-induced neutrophil flux and IL-1β production as compared with that in their non-transgenic littermates (Hu et al., 2010). These results indicate that TRIM30 is a negative regulator of NLRP3 mediated inflammatory responses.

#### Potential role of micro RNAs in mediating ROS-induced inflammasome activation

MicroRNAs serve as rheostats controlling NLRP3 activity. Bandyopadhyay et al. has investigated the involvement of miR-133a-1 in the activation of inflammasome (NLRP3) and IL-1β production (Bandyopadhyay et al., 2013). Mitochondrial uncoupling protein 2 (UCP2) is elevated following exposure to hyperoxia and miR-133a-1 is known to target UCP2. Suppression of UCP2 by siRNA enhances hydrogen peroxide (H_2_O_2_)-induced inflammasome activity, but overexpression of UCP2 decreases the inflammasome activation. This suggests that miR-133a-1 suppresses inflammasome by negatively regulating UCP2. The miR-223, a myeloid specific microRNA is another critical regulator of NLRP3 inflammasome activity. miR-223 suppresses NLRP3 expression through a conserved binding site within the 3' untranslated region of NLRP3, leading to reduced NLRP3 inflammasome activity (Bauernfeind et al., 2012). The link between ROS, microRNA and inflammasome activation is yet to be clearly elucidated.

#### Mechanisms of ROS-induced NLRP3 activation

**Figure 2:**
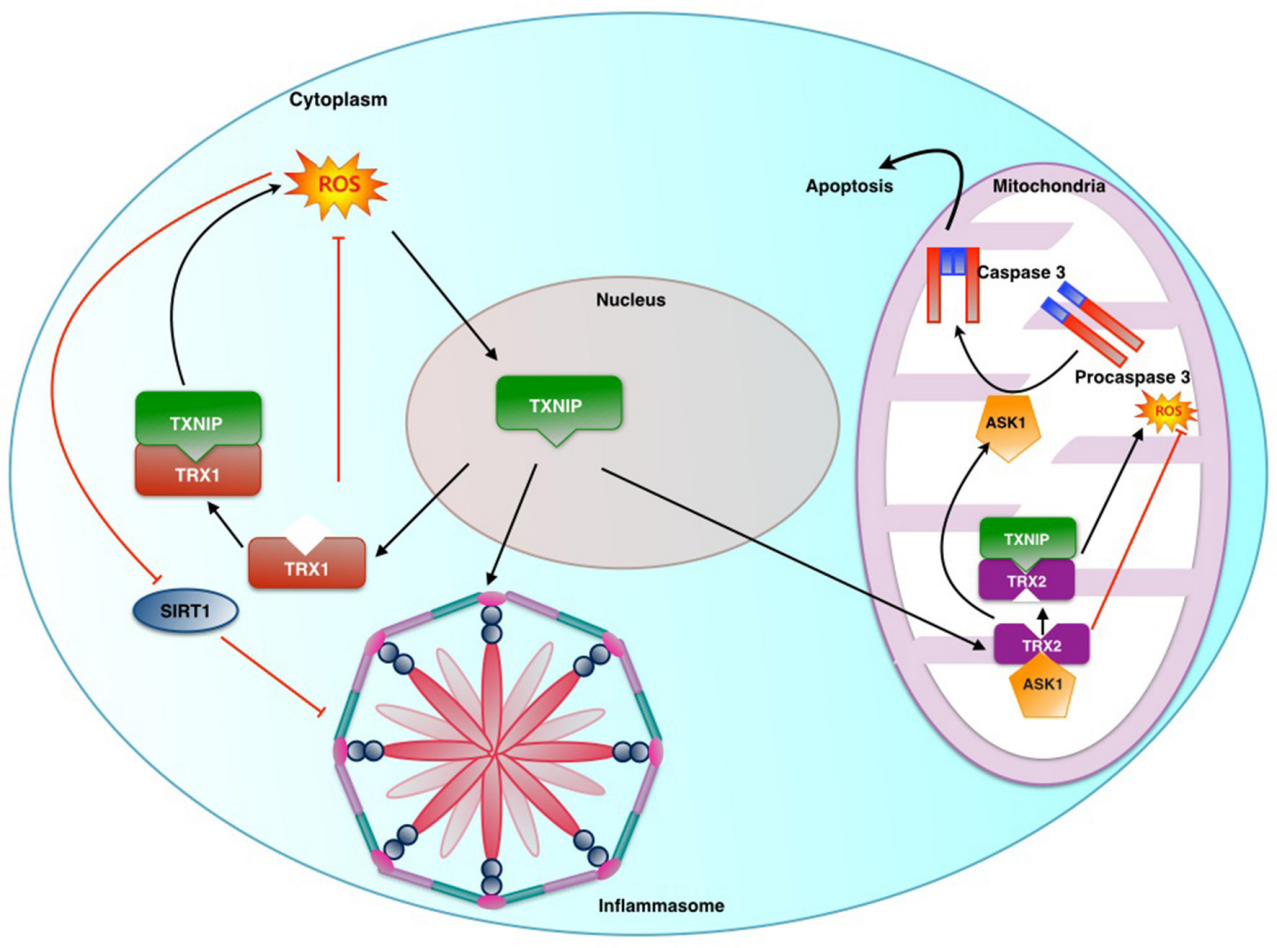
**Role played by Thioredoxin Interactive Protein (TXNIP) and Sirtuin in ROS induced inflammasome activation: Thioredoxin-1 (TRX1) and Thioredoxin-2 (TRX2) are part of key anti-oxidant systems in cytoplasm and mitochondria respectively**. Under stress TXNIP is transferred from its usual location of nucleus to mitochondria and cytosol. TXNIP in the nucleus binds to TRX2 uncoupling it from ASK1. This results in ASK1 mediated activation of caspase-3 and promotion of ROS production by virtue of removal of inhibitory action of TRX2. Similarly TRX1 action is affected in the cytosol.Sirt1 is an NAD+-dependent class III protein deacetylase which inhibits the transactivation potential of NF-κb by deacetylation. This causes suppression of NLRP3 transcription.

Several NLRP3 activators such as ATP, silica and asbestos trigger ROS generation and its suppression using ROS scavengers blocks NLRP3 activation. ROS is known to activate several members of the tyrosine kinases, and G-protein-coupled receptors. The downstream Raf kinase activates MEK1/2 MAP kinase, which in turn activates extracellular signal-regulated protein kinases 1 and 2 (ERK1/2) (Dhingra et al., 2007; Scholl et al., 2007). ERK1/2 directs the cell toward a pro- or anti-apoptotic state during inflammation. This is achieved by regulating expression of pro-inflammatory cytokines like TNF-α, IL-1β, and IL-18. A decrease in ROS production is accompanied by decreased ERK1/2 activation along with decreased IL-1β production (Cruz et al., 2007). ROS also activates inflammasome-activating signal transduction pathways via PI3K. Recent studies have brought attention to ROS-mediated activation of inflammasome via Thioredoxin (TRX) and its endogenous inhibitor Thiredoxin-interactive protein (TXNIP) (Zhou et al., 2010). High levels of TXNIP inhibit redox activity of cytoplasmic Thioredoxin-1 (TRX1) (Schulze et al., 2004; Yoshihara et al., 2014) as well as mitochondrial Thioredoxin-2 (TRX2) (Saxena et al., 2010). Oxidative stress promotes translocation of TXNIP from nucleus to mitochondria. TXNIP binds to TRX2, inhibiting its ability to keep ROS level under check. In addition, TXNIP uncouples Apoptosis signal-regulating kinase (ASK) 1-TRX2 binding. ASK1 that is liberated gets phosphorylated, thereby activating Caspase-3 and promoting apoptosis (Lane et al., 2013; Lu and Holmgren, 2014). TXNIP released from oxidized TRX directly binds to leucine-rich regions of NLRP3, leading to the formation of inflammasome (Figure 2). Further, ROS may directly activate caspase-1 and indirectly activate caspase-3 via ERK1/2. ERK1/2 activation by ROS is mediated by p53 as a result of the damage to DNA by ROS. The exact interactions between ERK1/2 and caspase-1 have not yet been delineated. During Libby Amphibole (LA)-induced inflammation, components of the NALP3 inflammasome are transcriptionally activated. LA-induced activation of inflammasome is known to contribute to fibrosis (Shannahan et al., 2012). ROS is reported to be required for purinergic P2X7 receptor (P2X7R)-mediated NALP3 inflammasome activation (Kishimoto et al., 1986; Solini et al., 2013). While the agonist of P2X7, ATP is known to enhance hyperoxia-induced inflammasome activation, its antagonist, oxidized ATP suppresses the same, as shown in human alveolar epithelial cells. It has been noted that hyperoxia induces K^+^ efflux through the P2X7 receptor, leading to inflammasome activation (Kolliputi et al., 2010). Suppressor of cytokine signaling-1 (SOCS-1) functions as a negative regulator in ROS-induced apoptotic responses by promoting ASK-1 degradation. SOCS-1 over-expressing mice are protected from hyperoxia-induced inflammation, which is associated with inactivation of inflammasome (Kolliputi and Waxman, 2009). This demonstrates a critical role for SOCS-1 in inflammasome-mediated inflammation. A model for NLRP3 activation called APANAREI-axis has been suggested in macrophages. APANAREI stands for “ATP-mediated P2X7-receptor Activation of NOX-induced ROS production, which activates ERK1/2 and thereby caspase-1 in the NLRP3-Inflammasome” (Kuiper, 2009). This includes the P2X7- receptor, NOX, ROS, ERK1/2, NLRP3, and caspase-1. In this model, ATP binds to P2X7, which up regulates NOX-PI3Kinase, Ca^2+^ fluxes, activating MAP-Kinases and ERK1/2. This involves caspase-1 activation in the inflammasome, resulting in cleaved IL-1β. NLRP3 activation by ROS via ERK1/2 in the APANAREI-axis is based on observational studies though the exact mechanisms of signaling downstream of ERK1/2 to caspase-1 are still unclear. Along with mitochondrial apoptotic signaling, NF-κ B serves as priming signal to activate NLRP3 inflammasome, thereby increasing production of IL-1β. ATP-induced mitochondrial dysfunction and apoptosis release oxidized mitochondrial DNA (mtDNA) into the cytosol which binds and activates the NLRP3 inflammasome. Macrophages lacking mtDNA have a dramatic decrease in IL-1β production, but that does not prevent apoptosis. Bcl-2, which is a known anti-apoptotic protein, inversely regulates mitochondrial dysfunction and NLRP3 inflammasome activation. Thus, binding of cytosolic oxidized mtDNA to the NLRP3 inflammasome serves as one of the pathways leading to apoptosis (Shimada et al., 2012).

#### Contradictory role of ROS in NLRP3 inflammasome activation

Although there is compelling evidence for the role of ROS in inflammasome activation, some studies suggest a contradictory role for ROS in inflammasome activation. Significantly higher levels of ROS are found in Superoxide Dismutase-1 (SOD-1)-deficient macrophages, as they fail to detoxify reactive superoxide species. These cells secrete relatively less amounts of active IL-1β in response to inflammasome stimulants (Meissner et al., 2010; van de Veerdonk et al., 2010). Similarly, SOD-1-deficient mice are profoundly resistant to endotoxin-mediated shock. The reason for this defective IL-1β cytokine response is that elevated ROS levels in SOD-1–deficient cells decrease the cellular redox potential, and reversibly oxidize and glutathionylate cellular components including caspase-1 (Meissner et al., 2008). These studies suggest that ROS can have an inhibitory effect on inflammasome activation. A recent study using pharmacologic ROS inhibitors and small hairpin RNA (shRNA) to knockdown p22^phox^ in human monocytic cell lines has shown that NOX activity and ROS stimulate rather than inhibit the activation of caspase-1 (Dostert et al., 2008). The exact reasons behind the observed contradictions over the role of NOX and ROS in NLRP3 inflammasome activation are not clear. These differences can be explained by species variations, differential regulation of monocytes and macrophages, differences in spatio-temporal localization of ROS, and the presence of functionally redundant NOX proteins. Further, it is interesting to note that while ROS is crucial for secretion of IL-1β via inflammasome activation, mice defective in ROS generation and patients with chronic granulomatous diseases (CGD) have a pro-inflammatory phenotype. Patients with CGDs have defective NOX2 activity and hence cannot generate NADPH-dependent ROS. Interestingly, no decrease in caspase-1 activation or secretion of IL-1β or IL-18 has been observed in primary CGD monocytes. Compared to monocytes from unaffected subjects, activation of CGD monocytes by uric acid crystals induces a four-fold higher level of IL-1β secretion in patients with CGD. This is accompanied by an increased caspase-1 activation compared with control cells. Inhibition of ROS activity in *in vitro* studies by DPI (diphenyleneiodonium chloride) reveals an inhibition of IL-1β gene expression, resulting in a decrease in IL-1β secretion. Presence of an inflammatory phenotype characterized by granulomas and inflammatory bowel disease occurring in CGD patients may be explained ironically by an absence of ROS aggravating alternate inflammatory pathways.

Neutrophils are known to play a significant role in acute inflammation by activating NOX and thereby promoting ROS generation. Studies show that IL-1β processing in human neutrophils is dependent on caspase-1, elastase, and proteinase 3. It has been shown that NOX2-deficient neutrophils not only activate caspase-1 but also express NALP3, indicating that ROS is neither required for inflammasome activation nor for its priming, as has been reported for macrophages (Gabelloni et al., 2013; Sokolovska et al., 2013). Recent advances have shown that NOX need not be the primary source of ROS production during inflammasome activation. Data show that the NLRP3 inflammasome/IL-1β secretion axis is a highly regulated inflammatory pathway which is susceptible to changes in mitochondrial and intracellular ROS, as well as overall mitochondrial dysfunction. Experiments have been conducted in an inflammation model of primary mouse peritoneal macrophages using mitochondria-targeted drugs (Jabaut et al., 2013). The drugs used to induce mitochondrial dysfunction are (1) antimycin A, which blocks electron flow at complex III, (2) carbonyl cyanide-p-trifluoromethoxyphenylhydrazone (FCCP), which uncouples mitochondrial oxidative phosphorylation, (3) MnTBAP, a superoxide dismutase mimetic that targets the mitochondria, and (4) DPI and Ebselen broad-spectrum antioxidants. It has been noted that MnTBAP and ebselen block IL-1β secretion when added before stimulation whereas DPI augments IL-1β secretion. These effects are independent of the intracellular or mitochondrial ROS levels. It has also been found that FCCP significantly sustains the association of the NLRP3 inflammasome complex. Mitochondria-targeted drugs increase IL-1β secretion independent of their impact on mitochondrial function and ROS levels. This suggests that both mitochondrial ROS-dependent and independent mechanisms play a role in the NLRP3 inflammasome/IL-1β secretion axis in serum amyloid A (SAA)-stimulated cells.

## Inflammasomes in human pathological conditions

Aberrations in inflammasomes have been implicated in the pathogenesis of several human diseases such as microbial infections, metabolic syndromes, organ injury and mucosal immune responses.

### Inflammasomes and microbial/viral infection

A vital role for NLRP3 inflammasome has been implicated in antibacterial, viral, fungal and parasitic immune responses; however, a direct link between NLRP3 inflammasome activation and the pathogen has been documented in only a few instances. Dengue is currently the most rapidly spreading mosquito-borne viral disease in the world, and the hemorrhagic form of the disease is a life threatening complication. Increased expression of IL-1β in platelets and platelet-derived micro particles is observed in patients with dengue or after platelet exposure to dengue virus *in vitro* (Hottz et al., 2013). Dengue virus infection leads to assembly of NLRP3 inflammasomes, activation of caspase-1 and caspase-1-dependent IL-1β secretion. Platelet-derived IL-1β is mainly released as microparticles through mechanisms dependent on mitochondrial ROS-triggered NLRP3 inflammasomes. Inflammasome activation and platelet shedding of IL-1β-rich microparticles correlates with increased vascular permeability (Hottz et al., 2013). These findings show that platelets contribute to increased vascular permeability in dengue virus infection by inflammasome-dependent release of IL-1β. Caspase-1 activation leading to up-regulation of pro-IL-1β, pro-IL-18, and NLRP3 is observed in dengue virus infected inflammatory macrophage (GM-M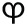). Blockade of CLEC5A/MDL-1, a C-type lectin critical for dengue hemorrhagic fever and Japanese encephalitis virus infection, inhibits NLRP3 inflammasome activation and pyrotopsis in GM-M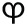. Dengue virus activates NLRP3 inflammasome via CLEC5A, and GM-M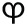 plays an important role in the pathogenesis of dengue infection (Wu et al., 2013b).

Human respiratory syncytial virus (RSV) causes life threatening respiratory diseases such as bronchiolitis and pneumonia in newborn, children, elderly, and immune-compromised individuals. Mechanism of activation of inflammation involves activation of TLR2/MyD88/NF-κB signaling, resulting in increased expression of pro-IL-1β and NLRP3 gene (Segovia et al., 2012). It is crucial that intracellular ROS and K^+^ efflux due to stimulation of ATP-sensitive ion channel promotes caspase-1-mediated inflammasome activation following RSV infection. In addition to NLRP3 inflammasome activation in viral infections, NLRP1 and NLRC4 inflammasome activation is driven by type III and type IV secretion systems (T3SS and T4SS) by *Salmonella, Pseudomonas, Legionella, and Yersinia* bacteria (Elinav et al., 2011). Secretion systems are transmembrane channels made up of large protein complexes present in the cell envelope of many bacteria through which proteins or protein–DNA complexes can be translocated (Wallden et al., 2010). Interestingly, to avoid inducing inflammasome activation, many bacteria encode inhibitors such as NS1 or Crm1, which directly modulate caspase-1 activity (Ray et al., 1992; Stasakova et al., 2005). Similarly, ORF63 of Kaposi's sarcoma-associated herpes virus binds NLR sensors and blocks formation of NLRP1 and NLRP3 inflammasomes (Gregory et al., 2011). Study of the role of inflammasomes in tuberculosis shows that the priming of primary murine microglial cells with conditioned media from cultures of macrophages infected with *Mycobacterium tuberculosis* (Mtb) results in activation of caspase-1 and IL-1β secretion (Dorhoi et al., 2012). Mtb-induced IL-1β maturation is found to depend on the generation of ROS and inflammasome activation. Dexamethasone, which is used as an adjunctive therapy to reduce inflammation in patients with tuberculous meningitis, significantly reduces the Mtb-induced maturation of IL-1β through inhibition of mitochondrial ROS generation (Lee et al., 2013). Future investigations need to address how pathogens are sensed by inflammasomes and how inflammasomes interact with innate immune pathways to regulate antimicrobial immune responses.

### Inflammasome activation by particulate and crystal compounds

In addition to microbial infectious agents, many non-infectious agents are known to induce inflammasome activation that is linked to pathologies in humans and animals. Endocytosis of inhaled silica and asbestos particles by residential lung macrophages results in increased ROS generation, lysosome destabilization and NLRP3 inflammasome activation, which leads to silicosis and asbestosis, respectively (Cassel et al., 2008; Hornung et al., 2008). Further, endogenous formation of crystals from monosodium urate (MSU) and calcium phosphate may result in inflammasome activation in macrophages. Accumulation of monosodium urate is characteristic of gout in humans, but only recently aberrant NLRP3 inflammasome activation, NLRP3-dependent caspase-1 activation, and release of IL-1β have been linked to its pathology. Impaired proteasomal degradation by MSU crystals enhances p62 expression which activates caspase-1 and IL-1β production. p62 is essential for activation of inflammasomes responsible for acute inflammation in gout (Amaral et al., 2012; Choe et al., 2014). Additionally, studies conducted using monosodium urate (MSU) crystals in murine dendritic cells (DC) from *Nlrp3^−/−^* mice suggest the role of inflammasomes in the disease process. DCs from Nlrp3^−/−^ mice respond differently to MSU or other ROS-mobilizing stimuli (rotenone and γ-radiation) compared to DCs from WT mice (Licandro et al., 2013). Furthermore, DNA fragmentation is markedly reduced in DCs from *Nlrp3^−/−^* and *Casp-1^−/−^* mice compared to those from WT mice. Another interesting example of activation of NLRP3 inflammasome by exogenous crystalline compound is by alum that is commonly used as an adjuvant in human vaccines. *In vitro* exposure of macrophages to alum leads to NLRP3-dependent activation of caspase-1 and NLRP3 inflammasome-deficient mice exhibit defects in alum-induced adaptive immune responses (Franchi and Núñez, 2008). However, other studies suggest that alum-induced adaptive immune responses may be mediated by ASC activation and recruitment independent of caspase-1 (McKee et al., 2009; Ellebedy et al., 2011). Alternate NLRP3-independent mechanism(s) for alum adjuvant effect has been proposed, which may involve attachment of the adjuvant to the plasma membrane of dendritic cells (Flach et al., 2011). These potential mechanisms of adjuvant-mediated inflammation remain to be fully defined.

### Inflammasome activation in diabetes

Inflammasomes may play a crucial role in pathobiology of diabetes. In macrophages, exogenous signals such as the saturated fatty acids activate the NLRP3 inflammasome and promote release of IL-1β. Fatty acid-activated AMP Kinase promotes phosphorylation of Unc-51 like kinase (ULK1), which initiates autophagy. The autophagy machinery controls mitochondrial homeostasis by removing old or damaged mitochondria. Fatty acids (e.g., palmitate) inhibit activation of AMP Kinase, leading to inhibition of autophagy. This results in accumulation of dysfunctional mitochondria, accompanied by enhanced generation of ROS. Increased generation of mitochondrial ROS stimulates activation of the NLRP3 inflammasome (Wen et al., 2011). One of the drugs used for glycemic control glyburide, a sulfonyl urea that stimulates insulin secretion, acts as an inhibitor of NLRP3 (Lamkanfi et al., 2009). Interestingly, therapeutic agents blocking IL-1α signaling improve both glycemic control of diet-induced obesity patients and pancreatic beta cell function in animal models (Osborn et al., 2008; Owyang et al., 2010). This suggests that the NLRP3 inflammasome is a promising therapeutic target in type 2 diabetes clinical trials. It has been shown that pancreatic islet amyloid polypeptide in diabetics activates NLRP3 inflammasome, as well as induces IL-1α secretion and inflammation of the islets, leading to the development of pancreatitis and deranged glycemic control (Masters et al., 2010). NLRP3 inflammasome activation is observed in chronic kidney disease and diabetic nephropathy. Local IL-1α secretion participates in the onset of kidney inflammation (Vilaysane et al., 2010). Atherosclerotic lesions containing abundant cholesterol crystals have also been shown to be a potent activator of NLRP3 (Duewell et al., 2010).

### Allergic contact dermatitis and inflammasome activation

Exposure to an allergen causes hypersensitivity and elicits a T cell-mediated inflammatory skin disease. Contact sensitizers activate the oxidative stress pathway in keratinocytes and dendritic cells (Corsini et al., 2013). ROS serves as essential second messenger in activating the NLRP3/NALP3 inflammasome mediating cellular responses, resulting in immune cells activation (Martin et al., 2011).

### Aging, oxidative stress, and inflammasome activation

Recent studies have revealed that a low-level oxidative stress is beneficial and can even extend the lifespan of organisms (Cadenas and Davies, 2000). ROS constitutes important signaling molecules required for autophagic degradation. Sirtuin1 (SIRT1), effectively acts against oxidative stress and can stimulate the expression of antioxidants via the FoxO pathway (Salminen et al., 2013). SIRT1 inhibits NF-κB signaling which is a major inducer of inflammatory responses via inflammasome pathway. ROS can inhibit SIRT1 activity by initiating oxidative modifications on its cysteine residues, and suppression of SIRT1 enhances the NF-κB signaling resulting in inflammatory responses, including inflammasome activation. Aging causes mitochondrial dysfunction with increased mitochondrial ROS, which could explain the link between enhanced mitochondrial ROS and inflammasome activation (Shimada et al., 2012).

### Hyperoxia, inflammasome activation, and acute lung injury

Oxygen therapy is an essential component in the management of severe lung diseases and in respiratory distress syndrome of preterm newborn. However, exposure to supra physiological concentration of oxygen could lead to hyperoxic acute lung injury (HALI), and *in vivo* studies have shown that HALI is suppressed in *Nlrp3^−/−^* mice compared with WT mice. *Nlrp3^−/−^* mice exposed to hyperoxia develop less injury compared to wild type mice, suggesting that HALI may be mediated through NLRP3 inflammasome (Fukumoto et al., 2013). Hyperoxia-induced recruitment of inflammatory cells and elevation of IL-1β, TNFα, macrophage inflammatory protein-2, and monocyte chemo attractant protein-1 are decreased in *Nlrp3^−/−^* mice. NLRP3 deletion attenuates lung epithelial cell death, caspase-3 levels and NF-κB levels compared to WT controls. These studies suggest a role for NLRP3 inflammasome activation in the development of hyperoxia-induced neonatal lung injury.

### Inflammasomes in autoimmune diseases

Systemic lupus erythematosus is characterized by an autoimmune response against nuclear antigen of oneself, especially the dsDNA. An increase in expression of the pro-inflammatory cytokine IL-1β has been noted in the cutaneous lesion and Peripheral Blood Mononuclear Cells (PBMCs) from lupus patients (Yang et al., 2014). IL-1β promotes a Th17 cell response, which is increased in lupus. Serum or purified IgG containing anti-dsDNA Abs along with self dsDNA activates NLRP3 inflammasome. ROS, along with K^+^ efflux, is involved in this activation. Inhibition of NLRP3, caspase-1, ROS, and K^+^ efflux decreases IL-1β production. It has also been noted that AIM2 expression is significantly increased in apoptotic DNA-induced macrophages and closely correlates with macrophage activation. Knockdown of AIM2 significantly blunts apoptotic DNA-induced macrophage activation. These findings provide new insights into the pathogenesis of lupus involving inflammasomes (Zhang et al., 2013).

### Inflammasomes and atherosclerosis

Accumulation of cholesterol in arterial plaques and IL-1β driven progression of atherogenesis are hallmarks of atherosclerosis. Recent microscopic evidence points to the presence of minute cholesterol crystals in early high fat diet-induced atherosclerotic lesions in LDL Receptor-KO mice, which coincides with the initial appearance of inflammatory cells in the lesions (Duewell et al., 2010). Further, this study demonstrates that cholesterol crystals activate NLRP3 inflammasome in resting or LPS-primed human PBMCs suggesting potential common mechanisms involved among the various crystals including monosodium urate. *In vivo* intraperitoneal administration of cholesterol crystals induces acute inflammation in WT mice, but not in mice deficient in components of NLRP3 inflammasome, cathepsin B, cathepsin L or IL-1 proteins. The most compelling evidence is obtained from LDL Receptor-KO mice reconstituted with NLRP3-KO bone marrow experiments wherein the aortic lesions are significantly smaller compared to mice reconstituted with wild-type bone marrow (Duewell et al., 2010). However, the possibility of oxidized-LDL-mediated activation of NLRP3 inflammasomes *in vivo* remains to be verified. Similar to human PBMCs, human macrophages also sense cholesterol crystals via NLRP3 inflammasomes (Rajamäki et al., 2010) suggesting NLRP3 as a therapeutic target in atherosclerosis. However, a recent study using the ApoE KO mice crossbred to NLRP3, ASC, or Caspase 1 KO mice shows no differences in atherogenesis or plaque stability to high fat diet, indicating that NLRP3 inflammasome may not play a role in atherogenesis in an ApoE-deficient mouse model (Menu et al., 2011). Interestingly, Nrf2-deficient ApoE KO mice are highly protected against high fat diet-induced atherogenesis, suggesting that Nrf2 is essential in driving cholesterol crystal-induced inflammation and exacerbation of atherosclerosis (Freigang et al., 2011). As ROS plays an important role in inflammasome activation, and atherosclerosis has been linked with mitochondrial dysfunction, it is reasonable to hypothesize that damage induced by high-fat diet or aging contributes to enhanced ROS production, which lowers the threshold required to trigger NLRP3 inflammasome activation (Madamanchi and Runge, 2007; Ordovas-Montanes and Ordovas, 2012). Further studies are necessary to take the above observations that NLRP3 inflammasomes may play a role in atherogenesis and progression of the disease from animal models to humans. The role played by inflammasomes in various pathological conditions and potential related therapeutic targets is summarized in Table 1.

**Table 1:** **Types of inflammasome activated in various human diseases and the potential therapeutic intervention**.

**Disease**	**Inflammasome activated**	**Potential related therapeutic intervention**
Dengue	NLRP3	Blockade of CLEC5A/MDL-1
Respiratory syncytial virus	NLRP3	Blockade of TLR2/MyD88/NF-κB signaling
Bacterial infection	NLRP3, NLRP1, NLRC4	Blockade of type III and type IV secretion systems
Silicosis, asbestosis, gout	NLRP3	Blockade of p62 in gout
Diabetes	NLRP3	Blockade of Unc-51 like kinase and IL-1α signaling
Allergic dermatitis	NLRP3, NALP3	–
Aging	NLRP3	Sirtuin1
Hyperoxic lung injury	NLRP 3	–
Autoimmune diseases	NLRP 3	Blockade of AIM2
Atherosclerosis	NLRP 3	Blockade of cathepsin B, cathepsin L

## Conclusion

Inflammasomes are multiprotein oligomers of the innate immune system that play a significant role in the pathogenesis of various diseases that include lung injury, dengue hemorrhagic fever, gout and diabetes mellitus. NLRP 3 and 6, NLRC4, and AIM2 are the prominent members of the inflammasome family which also includes precursor procaspase-1 and the adaptor ASC. Oxidant damage from ROS plays a significant role as an initiating process. Hyperoxia is also known to generate ROS which activates inflammasome sensor NLRP3. Activation of NLRP3 leads to activation of CLOCK gene, caspase 1 and further downstream targets such as IL-1β and IL-18, leading to activation of inflammatory cascade, pyroptosis and death of tissue. Various stimuli serve to activate inflammasome through the common activator ROS, but TRIM-30 and miR-133a-1 serve as suppressors of inflammasome activation. ROS activates inflammasome by MAP kinases and ERK1/2. Antioxidants such as SIRT-1 limit activation of NLRP3 inflammasome and confer protection from injury. Further research is required in understanding the mechanisms of activation of inflammasome. Inhibiting the activation of inflammasome may play a crucial therapeutic role for various pathological conditions involving activation of inflammasome.

### Conflict of interest statement

The authors declare that the research was conducted in the absence of any commercial or financial relationships that could be construed as a potential conflict of interest.
